# Evidence for host specificity of *Theileria capreoli* genotypes in cervids

**DOI:** 10.1186/s13071-017-2403-2

**Published:** 2017-10-10

**Authors:** Sándor Hornok, László Sugár, Gábor Horváth, Tibor Kovács, Attila Micsutka, Enikő Gönczi, Barbara Flaisz, Nóra Takács, Róbert Farkas, Marina L. Meli, Regina Hofmann-Lehmann

**Affiliations:** 1 Department of Parasitology and Zoology, University of Veterinary Medicine, Budapest, Hungary; 2 Department of Game Management and Ethology, Faculty of Agricultural and Environmental Sciences, University of Kaposvár, Kaposvár, Hungary; 3 Veterinary Authority, Csurgó, Hungary; 4 Veterinary Authority, Mórahalom, Hungary; 5 Food Chain Safety and Veterinary Authority, Pásztó, Hungary; 6 Clinical Laboratory and Center for Clinical Studies, Vetsuisse Faculty, University of Zurich, Zurich, Switzerland

**Keywords:** Buffalo, Red deer, Fallow deer, Roe deer, Wild boar, *Cervus*, *Capreolus*, *Theileria*

## Abstract

Data on the prevalence of piroplasms in buffaloes and large game animal species are lacking from several central European countries. Therefore, to investigate the presence of *Babesia*/*Theileria* DNA in these hosts, 239 blood and 270 spleen samples were taken from cervids (red, fallow, and roe deer), as well as from water buffaloes, mouflons, and wild boars in southwestern Hungary, followed by DNA extraction and molecular analysis for piroplasms. All samples from buffaloes and wild boars were PCR negative. Based on spleen samples, the prevalence of piroplasms was significantly higher in red deer (41.7%) than in fallow deer (23.5%). Two genotypes of *Theileria capreoli* were identified, which showed significant association with their host species (i.e. genotype “capreoli-CE1” was exclusively found in roe deer, whereas red and fallow deer harbored only genotype “elaphi-CE1”). Genotype “elaphi-CE1” of *T. capreoli* was also detected in one mouflon. No *Babesia* spp. were identified. In conclusion, in the evaluated region, genotypes of *T. capreoli* show host-associations among cervids, and at least one of these genotypes may infect mouflons.

## Letter to the Editor

In Europe, the estimated individual number of large game animal species, such as red deer (*Cervus elaphus*), roe deer (*Capreolus capreolus*), and wild boars (*Sus scrofa*) have been showing a steady increase during the past decades [1–3]. This also implies a tendency of growing the eco-epidemiological impact of relevant species. Apart from causative agents of infections that pose a threat only to cervids or wild boars, large game animals are also known to harbour pathogens that may affect domestic ungulates [4] or humans [5].

Ticks (Acari: Ixodidae) are regarded as the most important vectors (transmitters of pathogens) in the temperate zone [6]. Large game animal species are important hosts in the life-cycle of several tick species with high veterinary-medical significance, as exemplified by *Ixodes ricinus* [7] and *Haemaphysalis concinna* [8], thus influencing the dispersal and geographical distribution of relevant tick species [9].

Piroplasms (Apicomplexa: Piroplasmida) are tick-borne protozoa, infecting red and white blood cells of their vertebrate hosts [10]. Species, for which domestic and wild ungulates are susceptible, belong to *Babesia* (*sensu stricto*) and the genus *Theileria* [10], and in Europe, their most important tick vectors include *I. ricinus* and *Haemaphysalis* spp. [11, 12].

In Hungary, red deer and roe deer were shown to be important hosts of adults and immature stages of *I. ricinus* and *H. concinna* [13]. Also, several tick-borne pathogens have been reported from these tick species in the country, for which wild (game) animals are known to be reservoirs, including piroplasms [14]. However, relevant data, particularly molecular evidence on piroplasms infecting wild ungulates in Hungary are lacking.

Therefore, the aim of this study was to perform a molecular survey targeting piroplasms among water buffaloes (*Bubalus bubalis*) and individuals of five large game animal species: the red deer (*Ce. elaphus*), the fallow deer (*Dama dama*), the roe deer (*Ca.* *capreolus*), the mouflon (*Ovis orientalis*) and the wild boar (*S. scrofa*) in Hungary. The significance of these large game animal species is well illustrated by the fact that wild boars and cervids included in the present study occur in all countries of mainland Europe; mouflons are widespread in central and southern Europe; and the number of water buffalos living in Europe increased 1.5 times from 2000 to 2009 [4].

Samplings were performed between January 2013 and December 2014. In a natural reserve at Mórahalom (46°13′4.5″N, 19°53′1.3″E), blood samples were drawn from the jugular vein of water buffalos into EDTA tubes. These animals are kept extensively, grazing on grasslands from spring to autumn (thus exposed to ticks).

In an approximately 3000 km^2^ region of south-western Hungary (46°15′–46°50′N, 17°–17°50′E), blood or spleen samples were collected from game animals, depending on the time of death and conditions allowed by hunters. Blood samples were taken from the heart of fresh cadavers of game animals and transferred into EDTA tubes. If this was not possible, spleen sample was cut with sterile scalpel blade and put into plastic vial. Samples included in the study are shown in Table 1 (2nd, 3rd columns). All samples were frozen on the day of collection at -20 °C until processing.

**Table 1:** Results of molecular analyses of blood and spleen DNA samples from water buffalos and large game animal species in Hungary

Species	PCR positive/all tested samples (%)		No. of samples with *Theileria capreoli* genotype	
	Blood	Spleen	capreoli CE1	elaphi CE1
Water buffalo	0/60	–	–	–
Red deer	20/48 (41.7%)	40/96 (41.7)	–	33
Fallow deer	8/33 (24.2%)	20/85 (23.5)	–	20
Roe deer	28/65 (43.1%)	1/6 (16.7)	27	3
Mouflon	1/16 (6.3%)	0/4	–	1
Wild boar	0/17	0/79	–	–

The DNA was extracted individually, from 200 μl blood or *c.*10 mg of spleen with the QIAamp DNA Mini Kit (Qiagen, Hilden, Germany) according to the manufacturer’s instructions, including extraction controls to monitor cross-contamination of samples. All DNA samples were screened for the presence of piroplasms by a conventional PCR [15], which amplifies an approximately 500 bp fragment of the 18S rRNA gene of *Babesia*/*Theileria* spp. with the primers BJ1 (forward: 5′-GTC TTG TAA TTG GAA TGA TGG-3′) and BN2 (reverse: 5′-TAG TTT ATG GTT AGG ACT ACG-3′) as reported [16].

Each PCR was run with positive and negative controls (i.e. sequence-verified DNA of *Babesia canis*, and non-template reaction mixture, respectively). Negative controls and extraction controls remained PCR negative in all tests. Sanger-dideoxy sequencing was performed from all piroplasm PCR positive samples at Biomi Inc. (Gödöllő, Hungary). Only unambiguous sequences were considered further. Sequences were aligned and compared to reference GenBank sequences by nucleotide BLASTn program (https://blast.ncbi.nlm.nih.gov). Representative sequences were submitted to GenBank (accession number for *T. capreoli* genotype “capreoli-CE1”: KY308178; for genotype “elaphi-CE1”: KY308179).

Exact confidence intervals (CI) for the prevalences were calculated at 95% level. Prevalences were compared between identical sample types (blood or spleen) by Fisher’s exact test. Differences were regarded significant if *P* < 0.05.

Altogether 118 samples were PCR positive for piroplasms. All blood samples of buffalos and blood/spleen samples of wild boars were PCR negative. Based on spleen samples (Table 1), the prevalence of piroplasms was significantly higher in red deer (41.7%, CI: 31.7–52.2%) than in fallow deer (23.5%, CI: 15–34%) (*P* = 0.01). The PCR products from 84 positive samples were successfully sequenced, and in these only two genotypes of *T. capreoli* were identified. These two genotypes (designated here as “capreoli-CE1” and “elaphi-CE1”: Table 1) differed in one nucleotide (thymine vs adenine in position 681, respectively). Genotype “capreoli-CE1” was exclusively found in roe deer, whereas red and fallow deer harbored only genotype “elaphi-CE1” (Table 1). Thus, the occurrence of *Theileria* genotypes (“capreoli-CE1” vs “elaphi-CE1”) was strongly associated with host species (roe deer vs red deer and fallow deer: *P* < 0.0001).

In addition, genotype “elaphi-CE1” of *T. capreoli* was detected in one mouflon. No *Babesia* spp. were identified.

To the best of our knowledge, this is the first study in Europe, which demonstrated simultaneous and significant differences in the prevalences of *T. capreoli* genotypes among wild ruminant species in the same region. Here, genotype “capreoli CE1” was only found in roe deer, while genotype “elaphi CE1” was the only piroplasm detected in red deer and fallow deer. The observation that red and fallow deer share the same *T. capreoli* genotype, can be explained by the close taxonomic relationship of these ruminants (both in the Cervinae, but roe deer in the Capreolinae, see [17]). These results are in line with previous observations on the prevalences of theileriae in individual cervid species from other countries, i.e. *T. capreoli* identical with genotype “capreoli-CE1” of the present study was detected in four roe deer in Germany (KU510437 in [18]); *T. capreoli* identical with isolate “elaphi-CE1” of the present study predominated in red deer in Poland (*Theileria* sp. ZS-T04: DQ520836, see [19]) and fallow deer in Italy (GU373972 and GU373975, see [20]). The likely vector candidate for *T. capreoli* in Hungary is *H. concinna* because both above genotypes were found in questing nymphs and adults of this tick species in the country [14]. Furthermore, red deer and roe deer were shown to be important hosts of adults and immature stages of *H. concinna* in the same region of Hungary [13].

Genotype “elaphi-CE1” of *T. capreoli* was also detected in a mouflon. This host species has not been reported to be susceptible to this piroplasm. For instance, in a recent comprehensive survey carried out in Germany on piroplasms of game animals, only *Babesia* spp. were identified in mouflons [18].

Interestingly, no *Babesia* spp. were identified among sequenced PCR products in the present study. This finding contrasts with the moderate to high prevalence of *Babesia* spp. infection in large game animal species in nearby countries (i.e. 62.8% in roe deer in Germany: [18]; 10.7% in wild ruminants in Switzerland: [21]; 12.6% in roe deer in Italy: [22]). This discrepancy is most likely related to the game animals sampled in this study, which might have been different from those in other countries in the context of risk factors (host species, age, and altitude), shown to influence the prevalence of *Babesia* spp. in wild ruminants [21]. It is also highly relevant to consider that tick-infestation of wild ungulates may vary significantly between countries even within central Europe. For example, on roe deer *I. ricinus* (the vector of several *Babesia* spp. in Europe: [23]) was reported to predominate in Germany (i.e. with 92.6% of all ticks, see [24]), whereas *H. concinna* (the most likely vector of *T. capreoli*) represented 90.9% of all ticks collected from roe deer in Hungary [13].

In summary, in the study area*, T. capreoli* has two genotypes, which appear to be associated with particular species of cervids, and at least one of these genotypes is also able to infect mouflons.

## Abbreviation

CE: Central Europe

## References

[1] Burbaité L, Csányi S (2009). Roe deer population and harvest changes in Europe. Estonian J Ecol.

[2] Burbaité L, Csányi S (2010). Red deer population and harvest changes in Europe. Acta Zool Lit.

[3] Massei G, Kindberg J, Licoppe A, Gačić D, Šprem N, Kamler J (2015). Wild boar populations up, numbers of hunters down? A review of trends and implications for Europe. Pest Manag Sci.

[4] Martin C, Pastoret PP, Brochier B, Humblet MF, Saegerman C (2011). A survey of the transmission of infectious diseases/infections between wild and domestic ungulates in Europe. Vet Res.

[5] Yabsley M, Shock B (2013). Natural history of zoonotic *Babesia*: role of wildlife reservoirs. Int J Parasitol Parasites Wildl..

[6] Jongejan F, Uilenberg G (2004). The global importance of ticks. Parasitology.

[7] Medlock JM, Hansford KM, Bormane A, Derdakova M, Estrada-Peña A, George JC (2013). Driving forces for changes in geographical distribution of *Ixodes ricinus* ticks in Europe. Parasit Vectors.

[8] Nosek J (1971). The ecology, bionomics and behaviour of *Haemaphysalis* (*Haemaphysalis*) *concinna* tick. Z Parasitenkd.

[9] Hornok S, Mulvihill M, Szőke K, Gönczi E, Sulyok KM, Gyuranecz M, Hofmann-Lehmann R (2017). Impact of a freeway on the dispersal of ticks and *Ixodes ricinus*-borne pathogens: forested resting areas may become Lyme disease hotspots. Acta Vet Hung.

[10] Schreeg ME, Marr HS, Tarigo JL, Cohn LA, Bird DM, Scholl EH, et al. Mitochondrial genome sequences and structures aid in the resolution of Piroplasmida phylogeny. PLoS One. 2016:11, e0165702. 10.1371/journal.pone.0165702 PMC5104439 27832128

[11] Hunfeld KP, Hildebrandt A, Gray JS (2008). Babesiosis: recent insights into an ancient disease. Int J Parasitol.

[12] Mans BJ, Pienaar R, Latif AA (2015). A review of *Theileria* diagnostics and epidemiology. Int J Parasitol Parasites Wildl.

[13] Hornok S, Horváth G, Jongejan F, Farkas R. Ixodid ticks on ruminants, with on-host initiated moulting (apolysis) of *Ixodes*, *Haemaphysalis* and *Dermacentor* larvae. Vet Parasitol. 2012;187:350–3. 10.1016/j.vetpar.2011.12.012 22260900

[14] Hornok S, Takács N, Kontschán J, György Z, Micsutka A, Iceton S (2015). Diversity of *Haemaphysalis*-associated piroplasms of ruminants in central-eastern Europe, Hungary. Parasit Vectors.

[15] Casati S, Sager H, Gern L, Piffaretti JC (2006). Presence of potentially pathogenic *Babesia* sp. for human in *Ixodes ricinus* in Switzerland. Ann Agric Environ Med.

[16] Hornok S, Mester A, Takács N, Fernández de Mera IG, de la Fuente J, Farkas R. Re-emergence of bovine piroplasmosis in Hungary: has the etiological role of *Babesia divergens* been taken over by B. *major* and *Theileria buffeli*? Parasit Vectors. 2014;7:434. 10.1186/1756-3305-7-434 PMC4262194 25212410

[17] Fernández MH, Vrba ES (2005). A complete estimate of the phylogenetic relationships in Ruminantia: a dated species-level supertree of the extant ruminants. Biol Rev.

[18] Kauffmann M, Rehbein S, Hamel D, Lutz W, Heddergott M, Pfister K, Silaghi C (2017). *Anaplasma phagocytophilum* and *Babesia* spp. in roe deer (*Capreolus capreolus*), fallow deer (*Dama dama*) and mouflon (*Ovis musimon*) in Germany. Mol Cell Probes.

[19] Sawczuk M, Maciejewska A, Skotarczak B (2008). Identification and molecular characterization of *Theileria* sp. infecting red deer (*Cervus elaphus*) in northwestern Poland. Eur J Wildl Res.

[20] Galuppi R, Aureli S, Bonoli C, Caffara M, Tampieri MP (2011). Detection and molecular characterization of *Theileria* sp. in fallow deer (*Dama dama*) and ticks from an Italian natural preserve. Res Vet Sci.

[21] Michel AO, Mathis A, Ryser-Degiorgis MP. *Babesia* spp. in European wild ruminant species: parasite diversity and risk factors for infection. Vet Res. 2014;45:65. 10.1186/1297-9716-45-65 PMC4070358 24925474

[22] Zanet S, Trisciuoglio A, Bottero E, de Mera IG, Gortazar C, Carpignano MG, Ferroglio E (2014). Piroplasmosis in wildlife: *Babesia* and *Theileria* affecting free-ranging ungulates and carnivores in the Italian Alps. Parasit Vectors.

[23] Andersson MO, Bergvall UA, Chirico J, Christensson M, Lindgren PE, Nordstrom J, Kjellander P (2016). Molecular detection of *Babesia capreoli* and *Babesia venatorum* in wild Swedish roe deer, *Capreolus capreolus*. Parasit Vectors.

[24] Vor T, Kiffner C, Hagadorn P, Niedrig M, Rühe F (2010). Tick burden on European roe deer (*Capreolus capreolus*). Exp Appl Acarol.

